# Efficacy of low-dose hCG on FET cycle in patients with recurrent implantation failure

**DOI:** 10.3389/fendo.2022.1053592

**Published:** 2022-11-23

**Authors:** Xinyu Zhai, Mingming Shu, Yiming Guo, Shun Yao, Yiran Wang, Shujie Han, Chunlan Song, Yunhai Chuai, Qihang Wang, Fu Ma, Fu Chen, Ming Zhou, Wei Shang

**Affiliations:** ^1^ Navy Clinical Medical School, Anhui Medical University, Hefei, China; ^2^ The Fifth School of Clinical Medicine, Anhui Medical University, Hefei, China; ^3^ Department of Obstetrics and Gynecology, The Sixth Medical Center of Chinese PLA General Hospital, Beijing, China; ^4^ Department of Obstetrics and Gynecology, The Seventh Medical Center of Chinese PLA General Hospital, Beijing, China; ^5^ Department of Obstetrics and Gynecology, The First Medical Center of Chinese PLA General Hospital, Beijing, China; ^6^ Department of Biology, Kenneth P. Dietrich School of Art and Science, University of Pittsburgh, Pittsburgh, PA, United States; ^7^ Department of Obstetrics and Gynecology, First Hospital of Tsinghua University, Beijing, China

**Keywords:** human chorionic gonadotropin, recurrent implantation failure (RIF), whole exome sequencing (WES), functional enrichment analysis, gene mutation

## Abstract

**Objective:**

To study patients’ new treatment methods and mechanisms of repeated implantation failure.

**Design:**

A retrospective study.

**Setting:**

*In vitro* fertilization (IVF) unit in a Three-A hospital.

**Patient(s):**

Ninety-three patients with repeated implantation failure in IVF and embryo transfer.

**Intervention(s):**

the luteal phase support.

**Main outcome measure(s):**

According to whether human chorionic gonadotropin(HCG) was added, the two groups were divided into an observation group and a control group, and the clinical outcomes of the two groups were compared. Furthermore, 20 patients were selected for whole exome sequencing to investigate the mechanism.

**Results:**

The observation group’s clinical pregnancy rate and live birth rate were significantly higher than those in the control group (*P=0.004*). Functional enrichment analysis showed that these genes were significantly enriched in embryo implantation or endometrial receptivity processes, such as microtubule-based movement, NABA CORE MATRISOME, superoxide anion generation, protein localization to vacuole, extracellular matrix organization, fertilization, microtubule-based transport, cell junction organization, microtubule cytoskeleton organization. Furthermore, variants detected in these pathway genes were missense mutations that affect the protein’s biological activity but do not effectuate its inactivation.

**Conclusions:**

Adding HCG in the luteal phase might improve the clinical pregnancy and live birth rates in RIF patients. The potential pathogenesis of RIF genetic level may be caused by microtubule-based movement, extracellular matrix organization, and the Superoxide Anion generation pathway.

## Introduction

Repeated implantation failure (RIF) is a specific type of disease in assisted-reproductive therapy (1). Although there are many reports about RIF, uniform diagnostic criteria are not established (2). Orvieto et al. (3) and Zeyneloglu and Onalan (4) state that RIF is the disability to obtain a clinical pregnancy after three consecutive cycles of high-quality embryo transplantation. Koot et al. believe that gene expression profiles containing more than 300 genes can accurately predict and distinguish patients with repeated implantation failure (5). Haouzi D et al. found five potential genes to evaluate endometrial receptivity (6). Wagnini LD et al. found that the AA genotype of ESR1 and GT genotype of LIF were more common in RIF patients (7). Current clinical approaches to RIF include improving embryo quality and endometrial receptivity. Treatment methods to improve embryo quality include PGT technology, PGT-A, ZIFT and DFI reduction, *in vitro* fertilization (IVF), regimen change, and adjuvant drug treatment. The methods to improve endometrial receptivity include hormonal drug therapy (estrogen, progesterone, and bromocriptine Ting), adjuvant drug therapy (vitamin D, aspirin, low molecular weight heparin, microbial agents, and antibiotics), immunotherapy (prednisone, active immunity, passive immunity, and granulocyte colony-stimulating factor), assisted-reproductive therapy (intrauterine membrane opening period detection), surgical treatment (hysteroscopy and laparoscopy), Traditional Chinese medicine(TCM) treatment, stem cell treatment, and psychological treatment (8). These methods can treat some RIF patients; However, there is still a lack of effective treatment for some patients with RIF of unknown origin.

## Materials and methods

### Patients

The patients were generally in good condition, with no history of immunization, chromosomal abnormalities, and recurrent miscarriages. Ninety-three patients with RIF from the Sixth Medical Central of People’s Liberation Army of China General Hospital were analyzed. All patients signed the informed consent. The characteristics of the patients were as follows: (1) Repeated transplantation failure for unknown reasons; (2) Patients aged ≥35 years and < 45 years; (3) All patients were selected for statistical analysis in the last thawing transplant cycle; (4) The ovulation induction program for the cleavage embryos transplanted during the last thawing cycle was all a micro-stimulation program; (5) All endometrial preparation protocols for thawing transplant cycles were natural cycles. The patients were divided into observation and control groups according to different support methods in luteal support therapy. The observation group was treated with low dosage HCG combined with conventional luteal support therapy. In contrast, the control group was treated with only conventional luteal support therapy.

Whole exome sequencing analyses were conducted to understand the molecular mechanism of specific patients being responsive for low dose HCG treatment,. Ten patients with successful pregnancy from the observation group were used as case; Ten patients with unsuccessful pregnancy from the observation group were used as control. Blood samples for sequencing were obtained when the patients were not pregnant.

Approval was obtained from the Ethics Committee of the sixth medical center of PLA General Hospital before starting the study, all methods are implemented in accordance with relevant standards and regulations, and all patients have signed informed consent.

### Treatments

Observation group luteal phase support: Endometrial transformation (All cycles were frozen embryo transfer cycles) of the patients began to use Fenmaton (yellow tablets, Estradiol 2mg, Dydrogesterone 10mg), Progesterone soft capsules, and prednisone on the same day. The dosages were as follows: Fenmaton (yellow tablets, Estradiol 2mg, Dydrogesterone 10mg) 1 tablet three times/day per os (PO), Progesterone soft capsules 0.2g two times/day per vagina (PV), aspirin 25mg three times/day PO, prednisone 5 mg once/day PO, and ovulation D2 (D0 on ovulation day) three hormones (E_2_, LH, P; the dosage was adjusted according to D2 estrogen and progesterone levels. Each patient was injected intramuscularly with 500 IU of HCG every alternate day from the day after transplantation. The remaining medication was the same as before. On day 14, after transplantation, the levels of HCG, E_2_, LH, and P were examined. When HCG was >50 mIU/mL, pregnancy was confirmed. Four hormones (HCG, E_2_, LH, and P) were reviewed every other day to check HCG’s doubling status promptly. The estrogen and progesterone medications were adjusted, hormones were detected once a week for stability, and the frequency of follow-up visits was increased when hormones were unstable. Patients underwent vaginal ultrasounds 30–35 days after transplantation to check for intrauterine pregnancy and embryonic development. Abdominal ultrasonography was performed on day 49 to check the development of the fetus, which if appropriate, would allow the gradual reduction of the administration of estrogen, progesterone, and HCG. All drugs were discontinued by 12 weeks. Obstetric consultation was performed, and the follow-up period was 1 year. When 5≤HCG ≤ 50 mIU/mL, the medication was continued, and the four hormones (HCG, E_2_, LH, P) were reviewed every other day. If the pregnancy was confirmed, fetal maintenance was performed. If the HCG value was not doubled, the drug was discontinued. When HCG ≤ 5 mIU/mL and the HCG value was not doubled, it was determined that the patient was not pregnant, and the drug was discontinued. The luteal phase support in the control group was the same, except that HCG was not administered. In addition, whole exome sequencing was detected in the case and control groups. Genomic DNA was extracted from peripheral blood for further processing and sequencing for each example.

### DNA extraction, library construction

Total DNA from peripheral blood was extracted using DNeasy Blood & Tissue Kit(QIAGEN). DNA degradation and contamination were tested using 1% agarose gel electrophoresis, and Qubit DNA Assay Kit with a Qubit 3.0 Fluorometer was used to measure the quality and quantity of the extracted DNA. The samples with total DNA above 0.6μg were used for the library construction. The Agilent SureSelect Human All Exon V6 kit was used to efficiently enrich human exome region DNA, generating sequencing libraries, and the operation was carried out according to the manufacturer’s instructions. After the construction of the library, Qubit 2.0 was used for preliminary quantification, and NGS3K/Caliper was used to detect the insert size of the library. After the insert size was consistent with the expectations (180-280bp), the qPCR method was used to accurately quantify the library’s effective concentration (3 nM) to ensure the library’s quality.

The original sequencing sequence contains low-quality reads with connectors, which can significantly interfere with subsequent information analysis. In order to ensure the quality of information analysis, raw reads must be finely filtered to obtain Clean reads, and subsequent analysis is based on clean reads.

The steps of data processing are as follows:

1. Remove reads with adapters;
2. Remove reads with a proportion of N(N indicates that base information cannot be determined) greater than 10%;
3. When the number of low-quality (less than 5) bases in single-ended reads exceeds 50% of the length ratio of the read, the paired reads should be removed.

The information of DNA-SEQ connectors (Adapter, Oligonucleotide sequences for TruSeqTM DNA Sample Prep Kits) is as follows:

5’Adapter:

5’-AATGATACGGCGACCACCGAGATCTACACTCTTTCCCTACACGACGCTCTTCCGATCT-3’

3’Adapter:

5’-GATCGGAAGAGCACACGTCTGAACTCCAGTCAC (8index)ATCTCGTATGCCGTCTTCTGCTTG-3’

### Whole exome sequencing and variant detection

Whole-exome sequencing was performed on Novaseq6000 (Illumina) in 150 bp paired-end reads according to the effective concentration of the library and the data output requirements of 10G of each sample. After raw read filtering, sequencing error rate check, the Phred score (Q20 and Q30) proportions, the amount of raw data, and the mapping rate were evaluated to determine whether the data was up to the standard (the average proportion of Q20 bases is more than 90%, the average proportion of Q30 bases is more than 80%, and the average error rate is less than 0.1%). The clean data obtained were aligned to a reference genome (GRCh37/hg19) by BWA (9). SAMtools were used for variant detection (10). Variant annotations were obtained by the ANNOVAR method (11).

The variant screening principle was as follows: 1) the variant consequence should be frameshift, stop-gained, or stop-loss, which are generally determined as loss of function mutation for the gene; 2) the allele frequency should be less than 0.01 based on the GnomAD database (https://gnomad.broadinstitute.org); 3) missense mutation being predicted as deleterious or damaging by SIFT and PolyPhen-2 software were also kept (12–14). Functional enrichment analyses of genes possessing the above mutations were conducted using DAVID bioinformatics resources (15, 16). We used the KEGG pathway and different categories of Gene Ontology with biological process, molecular function, and cellular component terms to investigate the functions of genes we are interested in. Modified Fisher’s exact test was applied to give a statistically significant measure (p-value) combined with multiple testing corrections of the Benjamini technique.

### Outcomes measures

The outcomes were recorded between two groups: ectopic pregnancy, clinical pregnancy, multiple pregnancy, abortion, and live birth. The clinical pregnancy rate is the primary therapeutic index. Since the observation group used HCG, the biochemical pregnancy outcomes of the two groups were not compared. Clinical pregnancy was defined as a gestational sac in the uterus observed by a B-ultrasound examination of the abdomen 30–35 days after transplantation. Live birth was defined as the delivery of one or more living infants after 20 completed weeks of gestation.

### Statistical analysis

Statistical analysis was performed on the data using SPSS version 21.0. Quantitative data consistent with normal distribution were expressed as mean ± standard deviation, and two independent samples t-tests were used for comparison. Quantitative data that did not conform to the normal distribution were expressed as the median, P25, and P75, and the rank-sum test was used for comparison. Qualitative data are expressed as a percentage and compared using the Chi-square test. *P*<0.05 was considered statistically significant. Non-parametric tests, Mann-Whitney test, and Fisher’s Exact test were used for statistical analysis of whole exome sequencing results. The Benjamini-Hochberg method was used for multiple test corrections.

## Results

### Low dosage HCG treatment obtained better clinical outcome compared with conventional method

**Figure 1:**
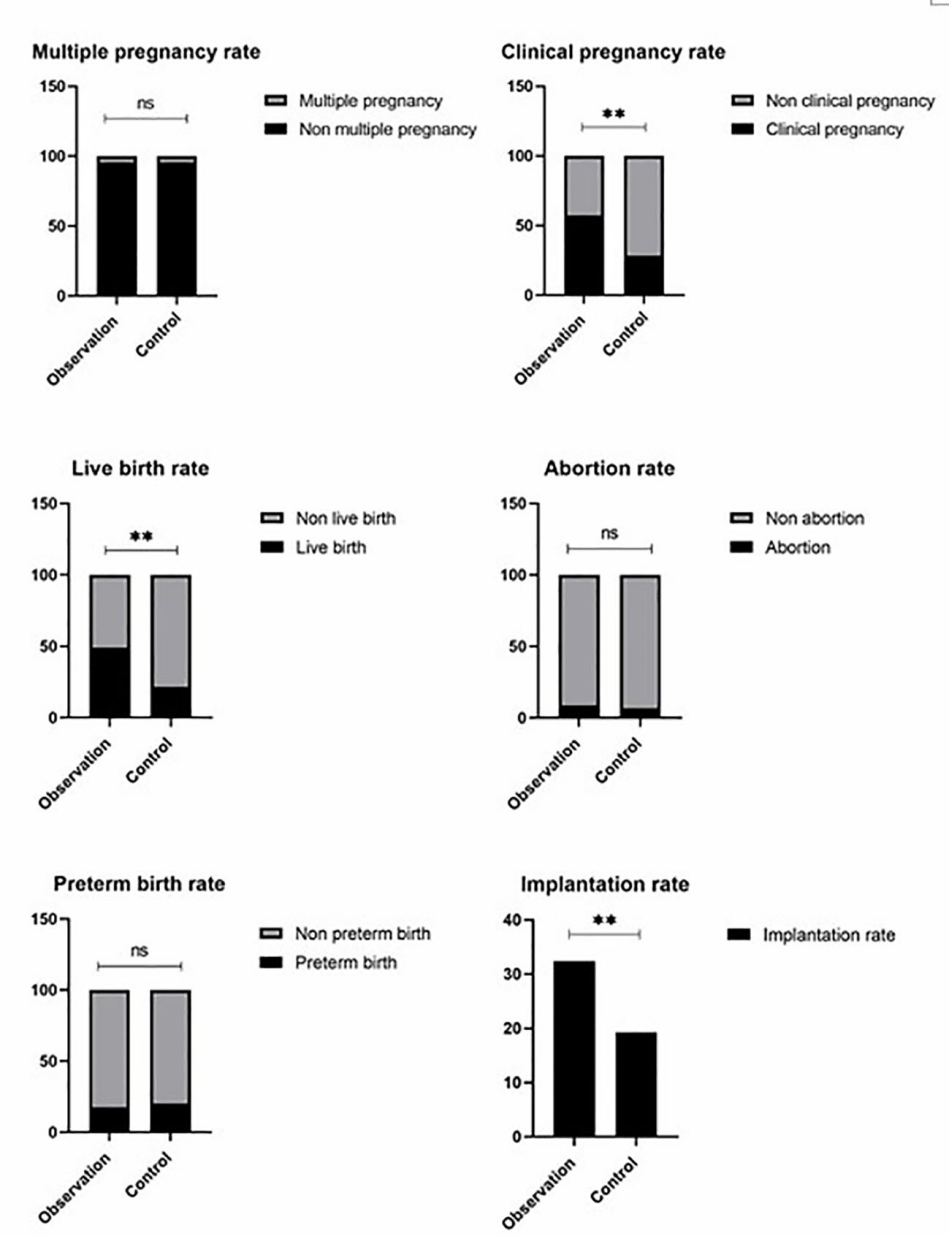
Observation group got signifiocantly better clinical pregnancy rate (57.4%vs28.3%, RR = 2.033, 95% CI: 1.206–3.426) and live birth rate 48.9%vs21.7%, RR = 2.251, 95% CI: 1.209–4.190) than control group (*P*< 0.05). There are no significant differences in the ectopic pregnancy rate of both groups was 0), multiple pregnancy rate, and abortion rate between the two groups (*P >*0.05). ns P > 0.05, ** P < 0.01.

The baseline of two groups is not biased (**Table 1**); no related adverse events were detected. We found no significant differences in the ectopic pregnancy rate (The ectopic pregnancy rate of both groups was 0), premature birth rate, multiple pregnancy rate, and abortion rate between the two groups (*P*>0.05) (**Table 2** and **Figure 1**). However, observation group got significantly better clinical pregnancy rate (57.4%vs28.3%, RR=2.033, 95% CI: 1.206–3.426) and live birth rate 48.9%vs21.7%, RR=2.251, 95% CI: 1.209–4.190) than control group (*P*<0.05) (**Table 2** and **Figure 1**).

**Table 1:** Comparison of basic situation between two groups of patients.

Group	Observation group	Control group	t or z or X^2^	*P*
Number	47	46		
Age (years) (`x±s )	39.81±3.24	39.24±2.80	0.907	0.367
BMI (KG/M2) (M, P25, P75)	21. 89, 20.50, 24.50	21.81, 20.42, 23.92	-0.461	0.645
Basal FSH (IU/L) ( M, P25, P75)	7.60,6.00,10.20	6.95,5.88,9.44	-1.064	0.287
Basal LH (IU/L) ( M, P25, P75)	3.20,2.39,4.20	3.00,1.98,4.47	-0.323	0.747
Basal E_2_ (pg/mL) (M, P25, P75)	39.00, 29.00, 50.20	40.75, 31.85, 48.63	-0.434	0.664
Basal P (ng/mL) ( M, P25, P75 )	0.44,0.34,0.63	0.45,0.29,0.59	-0.212	0.832
Infertility type, primary (%)	14 (29.8)	15 (32.6)	0.086	0.769
Infertility length (years) (M, P25, P75)	4, 3, 7	4, 2, 7	-0.229	0.819
Transformation day endometrial typing (number of individuals) A A-B B B-C C	4 (8.5) 13 (27.7) 12 (25.5) 8 (17.0) 10 (21.3)	5 (10.9) 7 (15.2) 13 (28.3) 10 (21.7) 11 (23.9)	2.210	0.697
Transplant day endometrial thickness (number) (M, P25, P75)	9.27±2.38	9.25±2.09	0.053	0.958
Number of transplanted embryos (number) (M, P25, P75)	2, 2, 3	2, 2, 2	-0.119	0. 905
Excellent embryo (number) (M, P25, P75)	2, 2, 2	2, 1, 2	-0.336	0.737

Observation group: HCG luteal support treatment given every other day after transplantation. Control group: HCG conventional luteal support is not given. P<0.05 was considered statistically significant.

**Table 2:** Comparison of clinical outcomes between two groups of patients.

Group	Observation group	Control group	X^2^	RR	95% CI	*P*
Number	47	46				
Implantation rate(%)	34 (32.4)	16 (15.5)	-2.942			0.003
Clinical pregnancy rate (%)	27 (57.4)	13 (28.3)	8.079	2.033	1.206-3.426	0.004
Live birth rate (%)	23 (48.9)	10 (21.7)	7.511	2.251	1.209-4.190	0.006
Premature birth rate (%)	4 (17.4)	2 (20.0)	0.032	0.870	0.189-4.001	0.858
Multiple pregnancy rate (%)	2 (4.3)	2 (4.3)	0.000	0.979	0.144-6.658	0.982
Abortion rate (%)	4 (8.5)	3 (6.5)	0.132	1.305	0.309-5.512	0.716

Observation group: HCG luteal support treatment given every other day after transplantation. Control group: HCG conventional luteal support is not given. P<0.05 was considered statistically significant.No ectopic pregnancy occurred in both groups.

### Patients with impaired genes involving in endometrium-embryo talk related pathways may be responsive to low dosage HCG treatment

**Figure 2:**
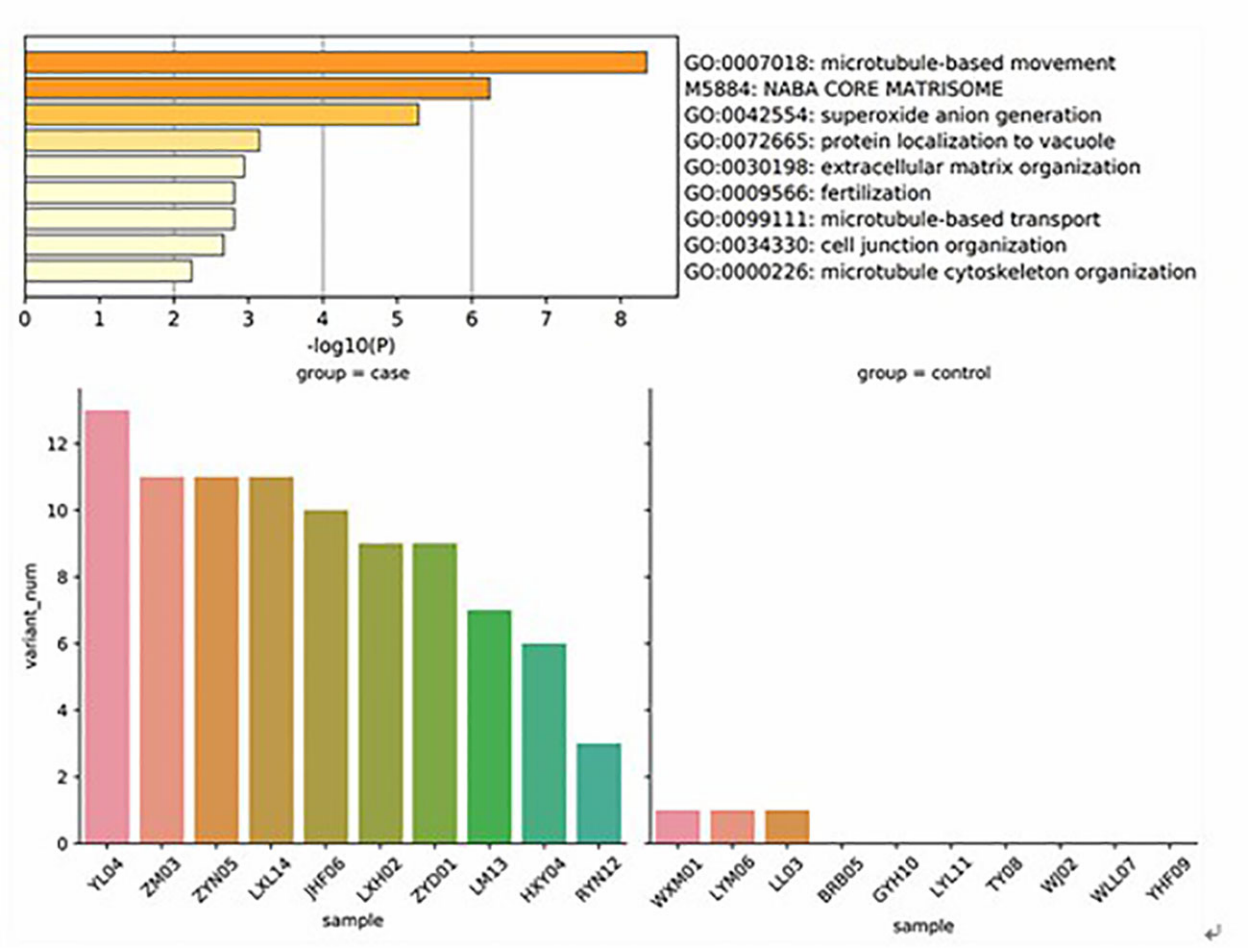
These genes were mainly enriched in microtubule based movement (GO: 0007018), NABA core matrix (M5884), superoxide anion generation (GO: 0042554), protein localization to vacuum (GO: 0072665), Extracellular matrix organization (GO: 0030198), utilization (GO: 0009566), microtubule-based transport (GO: 0099111), cell junction organization (GO: 0034330), microtubule cytoskeleton organization (GO: 0000226. It was found that the number of potentially harmful mutations carried by the case group was at least 5 loci and at most 13 loci; In the control group, only 3 samples carried a potentially harmful site, and the other 7 samples did not carry it.

**Figure 3:**
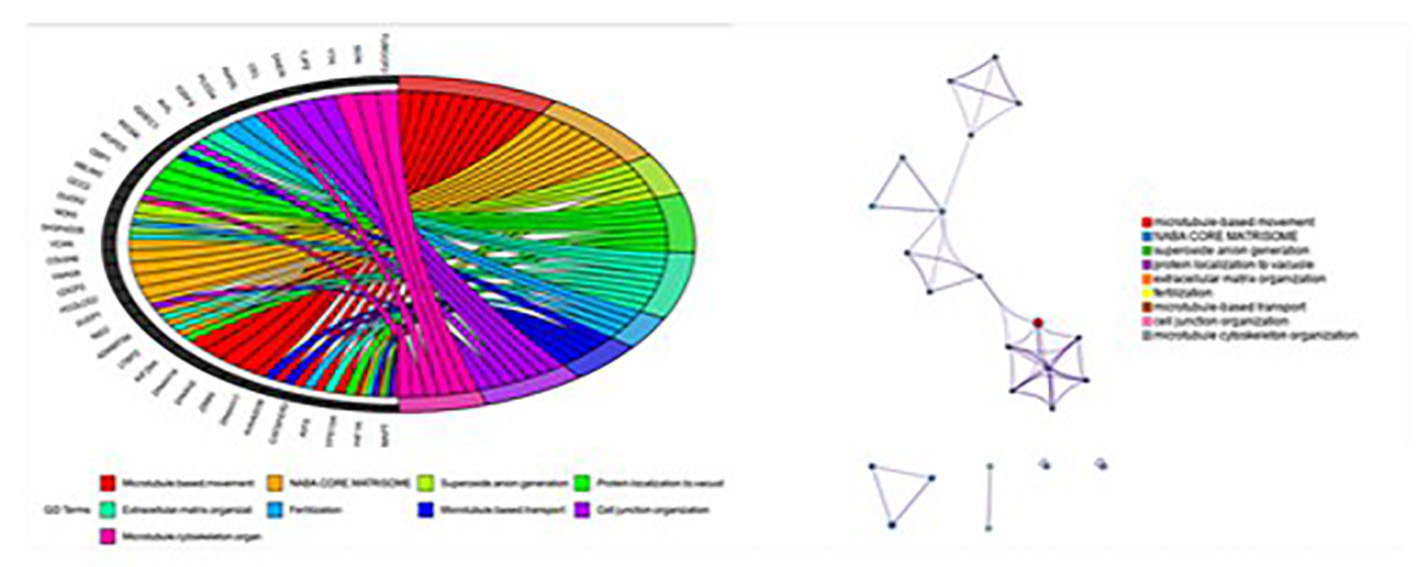
Goplot R package was used to visualize the relationship between these 39 genes and pathways; The Metascape method was used to conduct network enrichment analysis among pathways to study whether there was a relationship between the enriched pathways, and Cytoscape (https://cytoscape.org) software was used for visualization.

**Figure 4:**
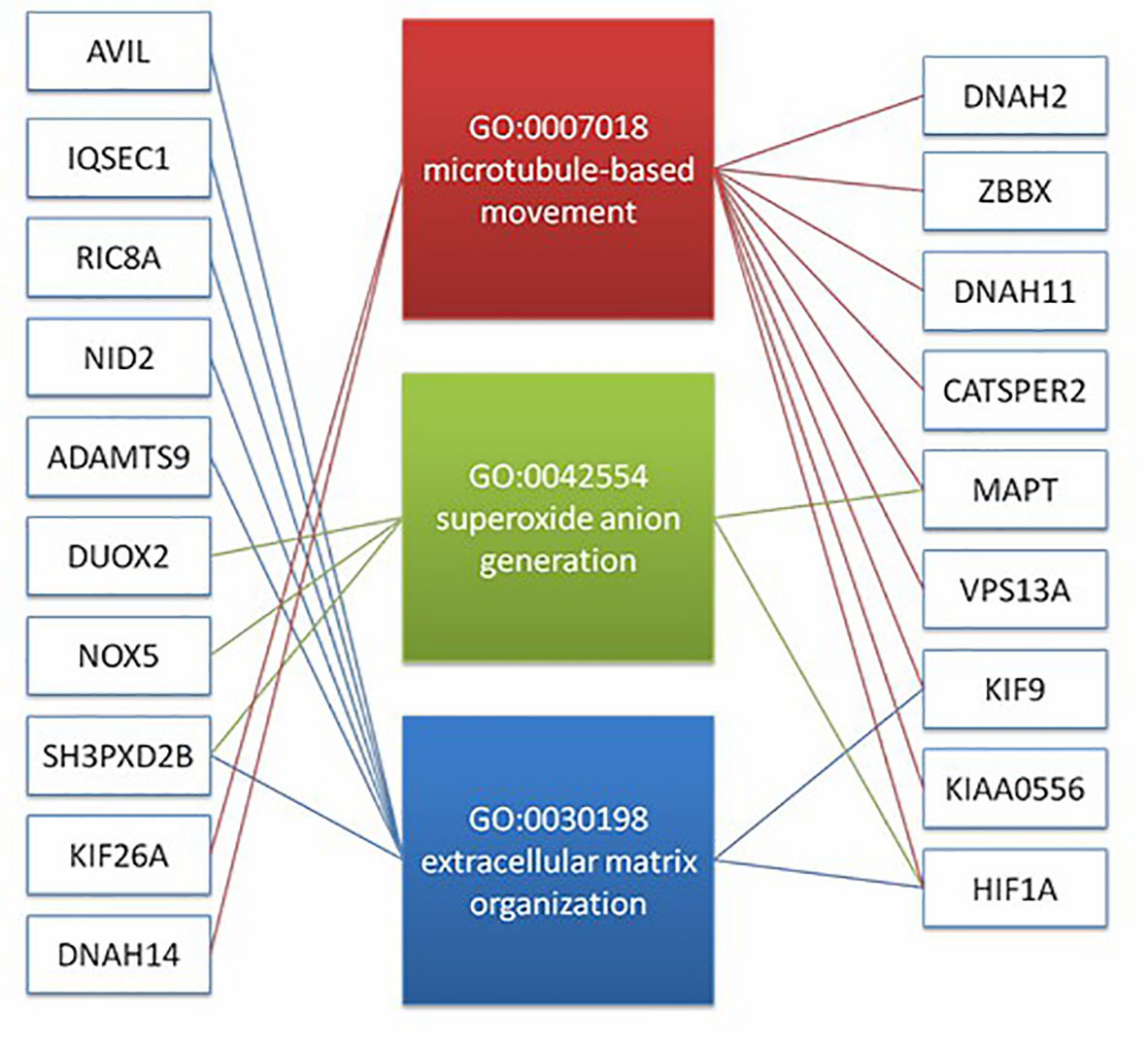
The result suggest that there may be a correlation between microtubule based movement, extracellular matrix organization, and superoxide anion generation pathway. It shows the diatribution of candidate genes on the three pathways (19 genes).

In order to understand the molecular mechanism of responsiveness of patients to low dosage HCG treatment, whole exome sequencing of patients’ peripheral blood was carried out. We explored the DNA mutation pattern of 10 patients with successful pregnancies and 10 with unsuccessful pregnancies after low dosage HCG treatment. We found that many genes with potential deleterious mutations (see Materials and Methods section) are detected in these patients. Due to the sample size, we did not screen the genes with a significantly higher frequency of harmful mutations in the case group. However, it may also be a potential information point for those genes with an increasing frequency of harmful mutations in the case group compared with the control group. For this reason, we use the analysis results of the occurrence frequency strategy of individual harmful mutation genes to select more than 2 individuals in the case group carrying specific gene harmful mutation sites (the control group should be ≤ 2 individuals). There is a difference of 2 or more individuals between the case group and the control group, such as GCC2 gene, and 5 individuals in the case group carrying harmful mutations of this gene. Only one individual in the control group carried this gene, which met our screening criteria. Based on this principle, we screened 73 genes. We used the Metascape method (17) for pathway enrichment analysis of these 73 genes. We found that they were mainly enriched in microtubule-based movement (GO: 0007018), NABA core matrix (M5884), superoxide anion generation (GO: 0042554), protein localization to vacuum (GO: 0072665), Extracellular matrix organization (GO: 0030198), utilization (GO: 0009566), microtubule-based transport (GO: 0099111), cell junction organization (GO: 0034330), microtubule cytoskeleton organization (GO: 0000226) (**Figure 2**). These nine pathways contain 39 genes. We studied the harmful mutations in the 39 candidate genes in the case group and the control group. It was found that the number of potentially harmful mutations carried by the case group was at least 5 loci and at most 13 loci; In the control group, only 3 samples carried a potentially harmful site, and the other 7 samples did not carry it (**Figure 2**). The Goplot R package was used to visualize the relationship between these 39 genes and pathways (18)(**Figure 3**). The Metascape method was used to conduct network enrichment analysis among pathways to study whether there was a relationship between the enriched pathways, and Cytoscape (https://cytoscape.org) software was used for visualization (**Figure 3**). The results suggested that microtubule-based movement, extracellular matrix organization, and Superoxide Anion generation pathway may be related (**Figure 4**). All these enriched functional categories involved in embryo implantation or endometrial receptivity processes. Furthermore, variants detected in these genes are mostly missense mutations which usually lead to a change in a particular amino acid of the protein. Being not loss-of-function mutations, like frameshift, stop-gained, and stop-loss, which result in protein inactivation, missense mutations only affect protein biological activity.

## Discussion

This study conducted a retrospective study on 93 patients with repeated transplantation failures. The clinical data of the patients were analyzed. When there was no significant difference in the age, body mass index, basic FSH level, basic E2 level, basic LH level, and basic p level between the two groups (P > 0.05), the implantation rate of the two groups was 32.4%vs15.5%. The implantation rate of the observation group was better than that of the control group, and the difference was statistically significant (P < 0.05). The clinical pregnancy rate of the two groups was 57.4%vs28.3%, and the live birth rate was 48.9%vs21.7%. The difference was statistically significant (P<0.05), and the premature birth rate (17.4%vs20.0%), ectopic pregnancy rate (no ectopic pregnancy in both groups), multiple pregnancy rate (4.3%vs4.3%), and the abortion rate (8.5%vs6.5%) was similar without significant difference (P > 0.05).

### A) Embryo implantation and luteal function

Embryo implantation is the key to mammalian reproduction. Successful implantation requires a good embryo and a synchronously developing endometrium, resulting from a series of complex molecular and genetic interactions. When the fertilized egg enters the uterus, it looks like a mulberry, that is, a mulberry embryo. The mulberry embryo continues to develop into blastocysts. A series of physiological changes (implantation window) also occur in the endometrium, including changes in gene expression, to prepare for embryo implantation. With the growth of the blastocyst, the zona pellucida dissolves. Under mechanical pressure and the peristalsis of uterine smooth muscle, the blastocyst is located on the endometrium, Endometrium in late secretory stage is transformed into endometrium in pregnancy under the action of hormones. This process is called decidualization (19). Decidualization requires the joint participation of a variety of substances. It is the result of a series of complex molecular interactions. It is the morphological and physiological changes of endometrium in order to adapt to pregnancy (20). Sex hormones such as estrogen, progesterone, transforming growth factors, interleukin family, signal transduction molecules, and immune cells are crucial for embryo implantation. These substances’ participation helps the embryo be successfully implanted into the endometrium (21).

After the follicle matures, it discharges the egg crown colliculus complex, some granulosa cells, and follicular fluid, with the follicle wall tension decreasing and collapsing inward, then the granulosa cells and thecal cells fill inward. Under the action of luteinizing hormone, luteinization forms two kinds of luteal cells, namely, granulosa luteal cells and thecal luteal cells. The corpus luteum is a gland formed after ovulation. The volume and function of the corpus luteum reach a peak 7-8 days after ovulation. The corpus luteum mainly undertakes the endocrine function of the ovary and is the primary source of estrogen and progesterone after ovulation. At the same time, the corpus luteum can also synthesize some protein hormones, such as oxytocin, inhibin, and relaxin, which play an essential role in embryo implantation and the maintenance of normal pregnancy. When the egg is not fertilized, the corpus luteum begins to degenerate 9-10 days after ovulation, and the function of the corpus luteum is limited to 14 days. However, the corpus luteum can be enlarged and transformed into the pregnant corpus luteum under the action of hCG, and it does not degenerate until the end of the third trimester of pregnancy (22). In this study, both groups of patients were prepared for the intima of a natural cycle. That is, both groups of patients would form corpus luteum after ovulation. The corpus luteum of the patients in the observation group was maintained with the support of hCG, the life of the corpus luteum was prolonged, and more estrogen, progesterone, and relaxin were secreted, which was conducive to embryo implantation. In the control group, the corpus luteum degenerated without hCG. Therefore, the continuous administration of small doses of hCG in the luteal phase increased the clinical pregnancy rate and live birth rate of the observation group, which may be related to the maintenance of the corpus luteum by hCG.

### B) HCG and endometrial receptivity

HCG is a glycoprotein hormone composed of two subunits secreted by syncytiotrophoblast cells β and α. Subunits are encoded by different genes on chromosomes 19 and 6, respectively α The subunit structure is the same as that of LH. LH is a kind of gonadotropin, secreted by basophils in the anterior pituitary gland. It is secreted in a pulsed manner under the impulse stimulation of gonadotropin-releasing hormones α and β, composed of two subunits. The role of LH includes promoting ovulation, luteal development, progesterone biosynthesis, and luteal maintenance. This pituitary gonadotropin induces the differentiation of follicular cells into steroid-derived luteal cells and further stimulates the synthesis of progesterone in luteal cells. HCG receptor and LH receptor are the same receptors, the hcg/lh receptor complex, also known as LHCGR (23). LHCGR is a G protein-coupled transmembrane receptor, which is expressed in reproductive organs and reproductive-related cells, such as luteal cells, endometrium, follicular granulosa cells, and chorion (24).. Due to hCG and LH β In terms of subunit structure, the half-life of hCG is much longer than that of LH, and the binding capacity of hCG and LHCGR is 6-8 times that of LH (25). HCG is more used to replace LH in clinical treatment. Typical signal pathways of LHCGR include stimulation of adenylate cyclase, which leads to the increase of intracellular cyclic adenosine monophosphate (cAMP), activation of protein kinase A (PKA) and phosphorylation of PKA substrate, and finally stimulates the production of prostaglandin E2 (PGE2). PGE2 can promote the proliferation, differentiation, and vascular penetration of endometrial stroma and glandular epithelial cells, promote endometrial remodeling and facilitate the localization and adhesion of early embryos in the endometrium (26). At the same time, the formation of neovascularization is critical in the process of endometrial hyperplasia. Vascular endothelial growth factor (VEGF) is the main factor to promote angiogenesis and participates in the reconstruction of blood vessels. VEGF exists in proliferative uterine fluid and the secretory phase of menstrual cycle. Some studies showed that the intrauterine VEGF level increased immediately after hCG (p<0.01). These results suggest that hCG may be involved in forming endometrial neovascularization (27). HCG can improve endometrial receptivity by acting on insulin-like growth factor binding protein-1 (IGFBP-1) and VEGF to stimulate endometrial angiogenesis (28). Meanwhile, hCG can inhibit the proliferation of uterine smooth muscle cells through the LHCGR receptor on the surface of endometrial smooth muscle cells, dynamically regulate the gap junction of smooth muscle cells, and reduce the contractility of uterine smooth muscle by changing the concentration of intracellular calcium ions, thus reducing the contractility of uterus and endometrial peristalsis wave, and helping embryo implantation (29). In addition, hCG also regulates the immune rejection at the maternal-fetal interface during the embryo implantation window (30). Some studies believe that hCG plays a role in regulating endometrial immune tolerance by regulating the balance between th1/th2 cells and inhibiting the maternal immune rejection of embryos (31). Some scholars have found that hCG can improve the immunosuppressive function of regulatory T cells by increasing the number of transcription factor Foxp3 (32). Other studies have shown that antagonists combined with hCG can increase the percentage of local cd56+cd16-nk cells in the endometrium of patients with repeated transplantation failure, thus reducing immune rejection and promoting embryo implantation in the endometrium (28). HCG can also affect the function of endometrial stromal cells and glandular epithelial cells through autocrine and paracrine, and then affect the secretion of cytokines and embryo implantation. In conclusion, hCG may promote embryo implantation through its effect on endometrial receptivity and immune regulation of window endometrium.

### C) Relaxin

Relaxin is a peptide hormone composed of two peptide chains with a relative molecular weight of about 6000. Its molecular structure is similar to that of insulin. Relaxin is mainly secreted by the granulosa luteal cells of the ovary, and a small part is secreted by the inner membrane cells of the follicle. Continuous administration of small doses of HCG to patients during the luteal phase maintained the function and longevity of the corpus luteum and was conducive to the secretion of relaxin. Some studies have found that relaxin and relaxin family peptide receptor 1 are expressed on granular luteal cells of monkeys and cats, suggesting that relaxin may regulate the function of granular luteal cells through autocrine (33). Stewart and other scholars found that the relaxin level secreted by granulosa luteal cells in the culture medium was positively correlated with embryo quality and embryo transfer rate. It suggests that the relaxin secreted by granulosa luteal cells may be involved in regulating embryo implantation and mitosis (34). The invasion of trophoblast into maternal decidual spiral artery mainly depends on matrix metalloproteinase, no system and natural killer cells. Relaxin can activate cyclooxygenase 1 and cyclooxygenase 2 and play an important role in angiogenesis, endometrial decidualization and initiation of implantation during embryo implantation (35). Relaxin is involved in regulating the balance between matrix metalloproteinase and tissue inhibitor of matrix metalloproteinase, significantly inhibiting the expression of matrix metalloproteinase in endometrium, increasing the expression of tissue inhibitor of matrix metalloproteinase, and promoting the structural change of decidual matrix (36). Relaxin can promote uterine spiral artery remodeling and endometrial angiogenesis by up-regulating VEGF (37). Relaxin can inhibit the release of platelets from local nuclear cells, reduce platelets aggregation, promote embryo invasion into intimal vessels, and reduce the release of anti-angiogenic factors such as thrombonectin (38, 39). Relaxin also selectively increases the content of local macrophages, natural killer cells, and neutrophils and induces maternal-fetal immune tolerance (40). HCG may stimulate the corpus luteum to maintain the function and longevity of the corpus luteum so that granular corpus luteum cells secrete a higher level of relaxin, thus promoting embryo implantation.

Based on the enriched pathways, 39 genes were selected, which are involved in microtubule-based movement, NABA CORE MATRISOME, superoxide anion generation, protein localization to vacuole, extracellular matrix organization, fertilization, microtubule-based transport, cell junction organization, and microtubule cytoskeleton organization. Most patients with successful pregnancies after low dosage HCG therapy have more than three mutations in these genes, and even one patient has thirteen mutations. In contrast, only three patients in patients with unsuccessful pregnancies were detected to possess one mutation in the target genes. The result may imply a higher probability of patients with target gene mutations getting good clinical outcomes when treated by low dosage HCG therapy. These genes may be potential biomarkers identified by low-dose hCG therapy targeting the RIF population.

Nevertheless, the present study has some limitations. The patients diagnosed with repeated implantation failure in our hospital from October 2014 to December 2017 were observed. Since this was a retrospective, single-center study, there was no prospective randomized controlled study. Furthermore, the small sample size of whole exome sequencing could cause interference in the observation results, and the endometrial preparation plan was not classified into sub-categories. In the future, the number of samples would be expanded to further explore whether such patients have the same gene mutation, elaborate the cause of subclinical hypophyseal hypofunction, and the underlying mechanism leading to RIF.

## Conclusions

The addition of HCG in the luteal phase might improve the clinical pregnancy rate and the live birth rate in RIF patients. The potential pathogenesis of the RIF genetic level may be caused by microtubule-based movement, extracellular matrix organization, and the Superoxide Anion generation pathway.

## Data availability statement

The datasets presented in this study can be found in online repositories. The names of the repository/repositories and accession number(s) can be found in the article/supplementary material.

## Ethics statement

The studies involving human participants were reviewed and approved by Chinese People’s Liberation Army General Hospital (The Sixth Medical Center of Chinese PLA General Hospital). The patients/participants provided their written informed consent to participate in this study. Written informed consent was obtained from the individual(s) for the publication of any potentially identifiable images or data included in this article.

## Author contributions

XZ, MS, and YG conceived the experiment, performed clinical manipulation and data analysis, and drafted the manuscript. SY and YW performed clinical treatments and recorded the data. SH, CS, and YC contributed to image processing. QW, FM, FC, and MZ were responsible for sequencing. WS supervised the study and reviewed and revised the critical content of the paper. All authors contributed to the article and approved the submitted version.

## Funding

The National Key Research and Development Program of China (grant 2018YFC1003003) supported this work.

## Acknowledgments

This study was not funded by other organizations and individuals.

## Conflict of interest

The authors declare that the research was conducted in the absence of any commercial or financial relationships that could be construed as a potential conflict of interest.

## Publisher’s note

All claims expressed in this article are solely those of the authors and do not necessarily represent those of their affiliated organizations, or those of the publisher, the editors and the reviewers. Any product that may be evaluated in this article, or claim that may be made by its manufacturer, is not guaranteed or endorsed by the publisher.

## References

[1] Bashiri AEA. Recurrent implantation failure-update overview on etiology, diagnosis, treatment and future directions. Reprod Biol Endocrinol (2018) 16:121. doi: 10.1186/s12958-018-0414-2 30518389 PMC6282265

[2] Simon A Laufer N. Assessment and treatment of repeated implantation failure (RIF). J Assist Reprod Genet (2012) 29:1227–39. doi: 10.1007/s10815-012-9861-4 PMC3510376 22976427

[3] Orvieto R Brengauz M Feldman B. A novel approach to normal responder patient with repeated implantation failures–a case report. Gynecol Endocrinol (2015) 31:435–7. doi: 10.3109/09513590.2015.1005595 25731193

[4] Zeyneloglu HB Onalan G. Remedies for recurrent implantation failure. Semin Reprod Med (2014) 32:297–305. doi: 10.1055/s-0034-1375182 24919029

[5] Koot YE van Hooff SR Boomsma CM van Leenen D Groot Koerkamp MJ Goddijn M. An endometrial gene expression signature accurately predicts recurrent implantation failure after IVF. Sci Rep (2016) 6:19411. doi: 10.1038/srep19411 26797113 PMC4726345

[6] Haouzi D ahmoud M K Fourar M Bendhaou K Dechaud H De Vos J. Identification of new biomarkers of human endometrial receptivity in the natural cycle. Hum Reprod (2009) 24:198–205. doi: 10.1093/humrep/den360 18835874

[7] Vagnini LD Renzi A Petersen B Canas MDCT Petersen CG Mauri AL. Association between estrogen receptor 1 (ESR1) and leukemia inhibitory factor (LIF) polymorphisms can help in the prediction of recurrent implantation failure. Fertil Steril (2019) 111:527–34. doi: 10.1016/j.fertnstert.2018.11.016 30611552

[8] Coughlan C Ledger W Wang Q Liu F Li TC. Recurrent implantation failure: Definition and management. Reprod Biomedicine Online (2013) 28. doi: 10.1016/j.rbmo.2013.08.011 24269084

[9] Li H Durbin R. Fast and accurate short read alignment with burrows-wheeler transform. Bioinf (Oxford England) (2009) 25:1754–60. doi: 10.1093/bioinformatics/btp324 PMC2705234 19451168

[10] Li H Handsaker B Wysoker A Fennell T Ruan J Homer N. The sequence Alignment/Map format and SAMtools. Bioinf (Oxford England) (2009) 25:2078–9. doi: 10.1093/bioinformatics/btp352 PMC2723002 19505943

[11] Wang K Li M Hakonarson H. ANNOVAR: functional annotation of genetic variants from high-throughput sequencing data. Nucleic Acids Res (2010) 38:e164. doi: 10.1093/nar/gkq603 20601685 PMC2938201

[12] Kumar P Henikoff S Ng PC. Predicting the effects of coding non-synonymous variants on protein function using the SIFT algorithm. Nat Protoc (2009) 4:1073–81. doi: 10.1038/nprot.2009.86 19561590

[13] Ng PC Henikoff S. SIFT: Predicting amino acid changes that affect protein function. Nucleic Acids Res (2003) 31:3812–4. doi: 10.1093/nar/gkg509 PMC168916 12824425

[14] Adzhubei IA Schmidt S Peshkin L Ramensky VE Gerasimova A Bork P. A method and server for predicting damaging missense mutations. Nat Methods (2010) 7:248–9. doi: 10.1038/nmeth0410-248 PMC2855889 20354512

[15] Huang da W Sherman BT Lempicki RA. Bioinformatics enrichment tools: paths toward the comprehensive functional analysis of large gene lists. Nucleic Acids Res (2009) 37:1–13. doi: 10.1093/nar/gkn923 19033363 PMC2615629

[16] Huang da W Sherman BT Lempicki RA. Systematic and integrative analysis of large gene lists using DAVID bioinformatics resources. Nat Protoc (2009) 4:44–57. doi: 10.1038/nprot.2008.211 19131956

[17] Zhou Y Zhou B Pache L Chang M Khodabakhshi AH Tanaseichuk O. Metascape provides a biologist-oriented resource for the analysis of systems-level datasets. (2019) 10:1523. doi: 10.1038/s41467-019-09234-6 PMC6447622 30944313

[18] Walter W Sánchez-Cabo F Ricote M. GOplot: an r package for visually combining expression data with functional analysis. Bioinf (Oxford England) (2015) 31:2912–4. doi: 10.1093/bioinformatics/btv300 25964631

[19] Chen X Man GCW Liu Y Wu F Huang J Li TC. Physiological and pathological angiogenesis in endometrium at the time of embryo implantation. Am J Reprod Immunol (2017) 78. doi: 10.1111/aji.12693 28466568

[20] Ashary N Tiwari A Modi D. Embryo implantation: War in times of love. Endocrinology (2018) 159:1188–98. doi: 10.1210/en.2017-03082 29319820

[21] Hamid HY Zakaria ZAB. Embryo implantation: Shedding light on the roles of ovarian hormones, cytokines and growth factors in the implantation process. Afr J Biotechnol (2012) 11(97):16297–304. doi: 10.5897/AJB12.1857

[22] Xie X Kong B Duan T. Obstetrics and gynecology [M] version 9 Vol. 19. . Beijing: People's Health Publishing House (2018).

[23] Ziecik AJ Kaczmarek MM Blitek A Kowalczyk AE Li X Rahman NA. Novel biological and possible applicable roles of LH/hCG receptor. Mol Cell Endocrinol (2007) 269(1-2):51–60. doi: 10.1016/j.mce.2006.08.016 17367919

[24] Pakarainen T Ahtiainen P Zhang FP Rulli S Poutanen M Huhtaniemi I. Extragonadal LH/hCG action–not yet time to rewrite textbooks. Mol Cell Endocrinol (2007) 269(1-2):9–16. doi: 10.1016/j.mce.2006.10.019 17350753

[25] Wei Z Gao M Zhang X. Research progress on the effect of human chorionic gonadotropin on the receptivity of endometrium. Reprod Contraception (2016) 36(9):758–62.

[26] Banerjee P Fazleabas AT. Endometrial responses to embryonic signals in the primate. Int J Dev Biol (2010) 54(2-3):295–302. doi: 10.1387/ijdb.082829pb 19876822 PMC5070538

[27] Licht P Russu V Wildt L. On the role of human chorionic gonadotropin (hCG) in the embryo-endometrial microenvironment: implications for differentiation and implantation. Semin Reprod Med (2001) 19:37–47. doi: 10.1055/s-2001-13909 11394202

[28] Filicori M Fazleabas AT Huhtaniemi I Licht P Rao ChV Tesarik J. Novel concepts of human chorionic gonadotropin: reproductive system interactions and potential in the management of infertility. Fertil Steril (2005) 84(2):275–84. doi: 10.1016/j.fertnstert.2005.02.033 16084861

[29] Mansour R Tawab N Kamal O El-Faissal Y Serour A Aboulghar M. Intrauterine injection of human chorionic gonadotropin before embryo transfer significantly improves the implantation and pregnancy rates in *in vitro* fertilization/intracytoplasmic sperm injection: a prospective randomized study. Fertil Steril (2011) 96(6):1370–1374.e1. doi: 10.1016/j.fertnstert.2011.09.044 22047664

[30] Gao M Zhang X Zhao L. Chinese Journal of reproduction and contraception. (2018) 38(1):57–9.

[31] Abu Alshamat E Al-Okla S Soukkarieh CH Kweider M. Human chorionic gonadotrophin (hCG) enhances immunity against l. tropica by stimulating Hum macrophage functions Parasite Immunol (2012) 34(10):449–54. 10.1111/j.1365-3024.2012.01368.x 22540351

[32] Horwitz DA Pan S Ou JN Wang J Chen M Gray JD. Therapeutic polyclonal human CD8+ CD25+ Fox3+ TNFR2+ PD-L1+ regulatory cells induced *ex-vivo* . Clin Immunol (2013) 149(3):450–63. doi: 10.1016/j.clim.2013.08.007 PMC3941976 24211847

[33] Bastu E Gokulu SG Dural O Yasa C Bulgurcuoglu S Karamustafaoglu Balci B. The association between follicular fluid levels of cathepsin b, relaxin or AMH with clinical pregnancy rates in infertile patients. Eur J Obstet Gynecol Reprod Biol (2015) 187:30–4. doi: 10.1016/j.ejogrb.2015.02.009 25739053

[34] Wang D Wang Y Qiao J. Biological role of relaxin in reproductive system [J]. Natl Med J China (2015) 95(25):2036–8.

[35] Baston-Büst DM Hess AP Hirchenhain J. A possible ambivalent role for relaxin in human myometrial and decidual cells *in vitro* . Arch Gynecol Obstet (2009) 280(6):961–9. doi: 10.1007/s00404-009-1046-8 19319551

[36] Cernaro V Lacquaniti A Lupica R Buemi A Trimboli D Giorgianni G. Relaxin: New pathophysiological aspects and pharmacological perspectives for an old protein. Med Res Rev (2014) 34(1):77–105. doi: 10.1002/med.21277 23401142

[37] Koos RD Kazi AA Roberson MS Jones JM. New insight into the transcriptional regulation of vascular endothelial growth factor expression in the endometrium by estrogen and relaxin. Ann N Y Acad Sci (2005) 1041:233–47. doi: 10.1196/annals.1282.037 15956714

[38] Foresta C Bettella A Vinanzi C Dabrilli P Meriggiola MC Garolla A. A novel circulating hormone of testis origin in humans. J Clin Endocrinol Metab (2004) 89(12):5952–8. doi: 10.1210/jc.2004-0575 15579743

[39] Shirota K Tateishi K Koji T Hishikawa Y Hachisuga T Kuroki M. Early human preantral follicles have relaxin and relaxin receptor (LGR7), and relaxin promotes their development. J Clin Endocrinol Metab (2005) 90(1):516–21. doi: 10.1210/jc.2004-0130 15483101

[40] Manaster I Mandelboim O. The unique properties of uterine NK cells. Am J Reprod Immunol (2010) 63(6):434–44. doi: 10.1111/j.1600-0897.2009.00794.x 20055791

